# Alarming resistance to third-generation cephalosporins and carbapenems among *Enterobacterales* colonising pregnant women and neonates in eastern DRC

**DOI:** 10.4102/ajlm.v15i1.2995

**Published:** 2026-09-01

**Authors:** Théophile Mitima Kashosi, Antonella Minutolo, Carlotta Fiorilla, Marialaura Fanelli, Daniella Ashuza Kagayo, Jean-Baptiste Kajiramugabi, Alfred Cubaka Kabagale, Sandro Grelli, Francesca Pica, Vittorio Colizzi

**Affiliations:** 1 Section Techniques de Laboratoire, Institut Supérieur des Techniques Médicales de Bukavu, Bukavu, Democratic Republic of the Congo; 2 Department of Microbiology and Virology, Doctoral School of Health Sciences and Technological Innovation, Evangelical University of Cameroon, Bandjoun, Cameroon; 3 Department of Experimental Medicine, Laboratory of Microbiology and Clinical Microbiology, Faculty of Medicine, University of Rome Tor Vergata, Rome, Italy; 4 Laboratoire de Microbiologie, Faculté des Sciences, Université Officielle de Bukavu, Bukavu, Democratic Republic of the Congo; 5 Virology Unit, Faculty of Medicine and Surgery, Policlinic of Tor Vergata, Rome, Italy; 6 Faculty of Medicine, Teaching Hospital ‘Good Samaritan’, N’Djamena, Chad

**Keywords:** extended-spectrum cephalosporin-resistant *Enterobacterales*, carbapenem-resistant *enterobacteriaceae*, multidrug resistance, pregnant women, newborns, Democratic Republic of the Congo

## Abstract

**Background:**

Extended-spectrum cephalosporin-resistant *Enterobacterales* (ESCR-E) and carbapenem-resistant *Enterobacteriaceae* (CRE) pose a growing threat to maternal and neonatal health, particularly in low-resource settings. In eastern Democratic Republic of the Congo (DRC), data on antimicrobial resistance in these populations are scarce.

**Objective:**

To determine resistance profiles of ESCR-E and CRE among pregnant women, postpartum mothers, and their newborns in South Kivu, eastern DRC.

**Methods:**

A cross-sectional multicentre study was conducted from April 2023 to October 2024 in urban and rural health facilities. Rectal swabs or stool samples were collected. Bacterial identification was performed using conventional biochemical galleries, and antimicrobial susceptibility testing was performed by the standard disk diffusion (Kirby–Bauer) method.

**Results:**

High rates of ESCR-E and CRE colonisation were found across all groups. Over 90% of ESCR-E isolates were resistant to third-generation cephalosporins and showed multidrug resistance, including to aminoglycosides and fluoroquinolones. Carbapenem-resistant *Enterobacteriaceae* isolates were resistant to penicillin and cephalosporins but remained susceptible to ceftazidime–tazobactam. These resistance profiles severely limit treatment options in maternal and neonatal care.

**Conclusion:**

The high prevalence of multidrug-resistant ESCR-E and CRE in mothers and newborns highlights an urgent need for improved antimicrobial stewardship, resistance surveillance, and infection prevention strategies. The potential use of probiotics to restore gut microbiota and reduce colonisation should also be explored.

**What this study adds:**

This study provides the first comprehensive data on ESCR-E and CRE colonisation in mothers and newborns in eastern DRC, revealing alarming multidrug resistance patterns and highlighting the urgent need for targeted stewardship, surveillance, and Infection Prevention and Control strategies in maternal and neonatal care settings.

## Introduction

Antimicrobial resistance (AMR) is a growing global public health crisis, threatening the effectiveness of modern medicine. In 2019, AMR was directly responsible for 1.27 million deaths and contributed to nearly 5 million, with the greatest burden in low- and middle-income countries.^1,2^ Sub-Saharan Africa is disproportionately affected because of fragile health systems, limited diagnostics, and poor antibiotic stewardship.^3^

Among the pathogens of highest concern are multidrug-resistant (MDR) *Enterobacterales*, particularly those resistant to third-generation cephalosporins (extended-spectrum cephalosporin-resistant *Enterobacterales*, ESCR-E) and carbapenems (carbapenem-resistant *Enterobacteriaceae*, CRE). These organisms significantly threaten maternal and neonatal health, limiting therapeutic options for both community- and hospital-acquired infections.^4^

Resistance to third-generation cephalosporins is commonly mediated by extended-spectrum β-lactamases (ESBLs) such as *Cefotaximase-Munich* (CTX-M), Temoniera (TEM), and sulfhydryl variable (SHV) types, while carbapenem resistance is often driven by carbapenemases such as *Bla*_NDM, *bla*OXA-48-like, and *blaKPC*.^5^ These genes are frequently carried on mobile genetic elements, facilitating rapid spread in both community and healthcare settings.^6^

Recent studies from sub-Saharan Africa report high faecal carriage of ESBL-producing *Enterobacterales* in pregnant women and neonates. Prevalence estimates range from 3.2% to over 60%, with hospitalisation and prior antibiotic use as key risk factors.^7,8,9^ In Tanzania, maternal ESBL carriage exceeds 60%, with isolates frequently resistant to fluoroquinolones and aminoglycosides.^10^ Similar trends in Uganda and Kenya link colonisation to subsequent neonatal sepsis, highlighting the clinical importance of these resistant organisms.^11^

Although CRE prevalence remains lower, their silent spread is increasingly reported. Regional carriage rates range from 1% to 4%, though this may be underestimated because of limited surveillance.^12^ Outbreaks in neonatal intensive care units, often involving multidrug-resistant Gram-negative organisms such as *Klebsiella pneumoniae* and *Enterobacter* spp., further illustrate the risks of nosocomial transmission.^13^

In eastern Democratic Republic of the Congo (DRC), particularly South Kivu, data on AMR are limited. A study at Bukavu General Hospital (2012–2013) reported high ESBL rates among *Enterobacterales*.^14^ Recent genomic analyses of *Escherichia coli* isolates from Bukavu identified international clones (e.g. ST131, ST405) harbouring CTX-M-15, and co-resistance to aminoglycosides and fluoroquinolones.^15^ In 2023, laboratory surveillance data from South Kivu reported that 31.4% of isolates were MDR or extensively drug-resistant, mainly involving *E. coli, K. pneumoniae* and *Enterobacter* spp.^16^ These findings underscore the persistently high burden of MDR strains in Bukavu and highlight the urgent need to investigate the underlying drivers and implement effective control strategies.^17^ Although local data on perinatal colonisation are scarce, studies in similar contexts report colonisation rates up to 90% in pregnant women and 64% in newborns, with isolates often resistant to carbapenems and other critical antibiotics.^18^

Understanding the resistance profiles of ESCR-E and CRE in maternal and neonatal populations is essential for guiding empirical therapy, infection control strategies, and antimicrobial stewardship, especially in resource-limited settings. Characterising resistance at the species level allows for targeted interventions where diagnostic capacity is limited.

However, data on maternal and neonatal rectal colonisation with ESCR-E and CRE in the DRC remain scarce. This evidence gap is critical because colonisation is a major precursor of early-onset neonatal infections, especially in settings with limited infection-prevention and control capacity. Strengthening local data is therefore essential to guide empirical therapy, neonatal sepsis prevention, and antimicrobial stewardship.

This study aims to describe the phenotypic resistance profiles of ESCR-E and CRE rectal isolates from pregnant women, postpartum mothers, and newborns in South Kivu, eastern DRC. By analysing susceptibility to a broad panel of antibiotics, this work provides crucial baseline data to inform both local and national health policies.

## Methods

### Ethical considerations

The study was approved by the National Health Ethics Committee of the DRC (certificate number CNES 001/DPSK/229PP/2023, 29 January 2023). Verbal informed consent was obtained after clearly explaining the objectives and procedures in the local language. A brief educational session on bacterial resistance and carriage of MDR bacteria during pregnancy was conducted beforehand to facilitate understanding and acceptance. Consent was given by participants or mothers before sampling. Participants’ confidentiality and anonymity were strictly maintained throughout the study. Data confidentiality was ensured by anonymisation and secure storage. The study complied with the Declaration of Helsinki, International Council for Harmonisation – Good Clinical Practice (ICH-GCP) E6(R2), and national regulations.

### Study design and setting

This descriptive, multicentre laboratory-based study took place from April 2023 to October 2024 across 10 health facilities in South Kivu province, eastern DRC. Five urban hospitals in Bukavu city were included: Panzi General Reference Hospital, Centre Hospitalier de la 8e Communauté des Églises de Pentecôte en Afrique Centrale de CAHI, Bukavu, Baptist Community Hospital Nyamugo, Institut Supérieur des Techniques Médicales de Bukavu-Bukavu Hospital Center, and Ciriri Hôpital Général de Référence. Five rural hospitals were also selected: Ciriri Hôpital Général de Références of Miti-Murhesa, Uvira, Walungu, Kaziba, and Nyantende, representing both urban and rural settings in a region affected by prolonged instability.

The study included pregnant women with a gestational age of 37 weeks or more who voluntarily attended antenatal consultation and agreed to participate after receiving health education on AMR and pregnancy. Exclusion criteria were pregnant women with known risk factors for bacterial colonisation, such as gestational diabetes, prolonged hospitalisation (> 10 days) during pregnancy, malignancy, and kidney and/or liver disease.

Sample size was calculated using the Kish–Leslie formula^19^: *n* = [*z*^2^ × *p* × (1–*p*)]/*e*^2^, where *n* is the minimum required sample size, *z* is the confidence level value (1.96 for 95% confidence), *p* is the estimated prevalence of ESBL carriage in pregnant women (64.3%, based on Tanzanian data^9^), and *e* is the margin of error (5%). The minimum sample size was estimated at 354 pregnant women. Convenience sampling was used, with consecutive recruitment of eligible pregnant women during their clinic visits until the sample size was reached.

Women identified as carriers of ESCR and/or CRE were followed until delivery, when fresh rectal swabs were collected from the mother and newborn, then sent again to the Institut Supérieur des Techniques Médicales de Bukavu laboratory and strains forwarded to Rome for confirmation.

### Sample collection and bacterial isolation

Rectal swabs were collected from pregnant women ≥ 37 weeks gestation after consent. Mothers identified as carriers at antenatal care had additional swabs taken at delivery from both them and their newborns. Samples were transported sterilely the same day to Institut Supérieur des Techniques Médicales de Bukavu.

Selective culture media used included CHROMagarTM ESBL (CHROMagar, Paris, France) for ESCR-E and CHROMagarTM mSuperCARBATM (CHROMagar, Paris, France) for CRE. Plates were incubated at 37°C for 18–24 h. Colonies with characteristic morphologies on CHROMagar were selected for further identification: on CHROMagar ESBL, ESBL-producing *E. coli* appeared as dark pink to reddish colonies; *Klebsiella* spp., *Enterobacter* spp., and *Citrobacter* spp. as metallic blue colonies, sometimes with a reddish halo; *Proteus* spp. as colonies with a brown halo; *Pseudomonas* spp. as cream translucent to blue colonies; and *Acinetobacter* spp. as opaque cream colonies. On CHROMagar mSuperCARBA, carbapenemase-producing *Enterobacterales* appeared as pink to mauve colonies. These isolates then underwent biochemical identification using oxidase tests, Kligler–Hajna agar, Simmons citrate, urea–indole medium, sulfide–indole–motility medium, lysine decarboxylase, and o-Nitrophenyl-β-D-galactopyranoside tests, as per established protocols.^20^

### Antimicrobial susceptibility testing

Antimicrobial susceptibility testing was performed by disk diffusion on Muller Hinton agar following European Committee on Antimicrobial Susceptibility Testing 2022 (Version 12.0) guidelines.^21^ For ESCR-E isolates, antibiotics tested included amoxicillin-clavulanic acid, ticarcillin, cefixime, ciprofloxacin, cefotaxime, ceftriaxone, ceftazidime, cefepime, gentamicin, amikacin, chloramphenicol, and tetracycline.

Carbapenem-resistant *Enterobacteriaceae* isolates were tested against the same antibiotics plus carbapenems: imipenem, meropenem, and ertapenem.

Zone diameters were interpreted using European Committee on Antimicrobial Susceptibility Testing 2022 breakpoints.^21^ Multidrug resistance was defined as resistance to at least one agent in three or more antimicrobial classes. The bacterial species were identified by biochemical characteristics using the conventional biochemical gallery.^22^

### Data processing and analysis

Bacteriological data were entered into a standardised database. Descriptive statistics, including frequencies and percentages of resistance, were computed using Epi Info 7.2.5.0. Resistance profiles were stratified by bacterial species (e.g. *E. coli, K. pneumoniae, Acinetobacter baumannii*), and patient group (pregnant women, delivery mothers, newborns). No inferential statistics were performed as the study was descriptive.

## Results

### Prevalence of faecal carriage of extended-spectrum cephalosporin-resistant *Enterobacterales* and carbapenem-resistant *Enterobacteriaceae* in pregnant and delivery women and newborns

Figure 1 shows the prevalence of faecal carriage of ESCR-E and CRE among pregnant women, women at delivery, and newborns. High rates of ESCR-E carriage were observed in all groups: 94.9% (336/354) in pregnant women, 100% (322/322) in women after delivery, and 83.90% (276/329) in newborns. Carbapenem-resistant *Enterobacterales* carriage was also high compared with the African average, with 25.1% (89/354) in pregnant women, 28.88% (93/322) in women at delivery, and 6.07% (20/329) in newborns. These results indicate a substantial faecal carriage of MDR *Enterobacterales* in both mothers and their newborns.

**FIGURE 1:**
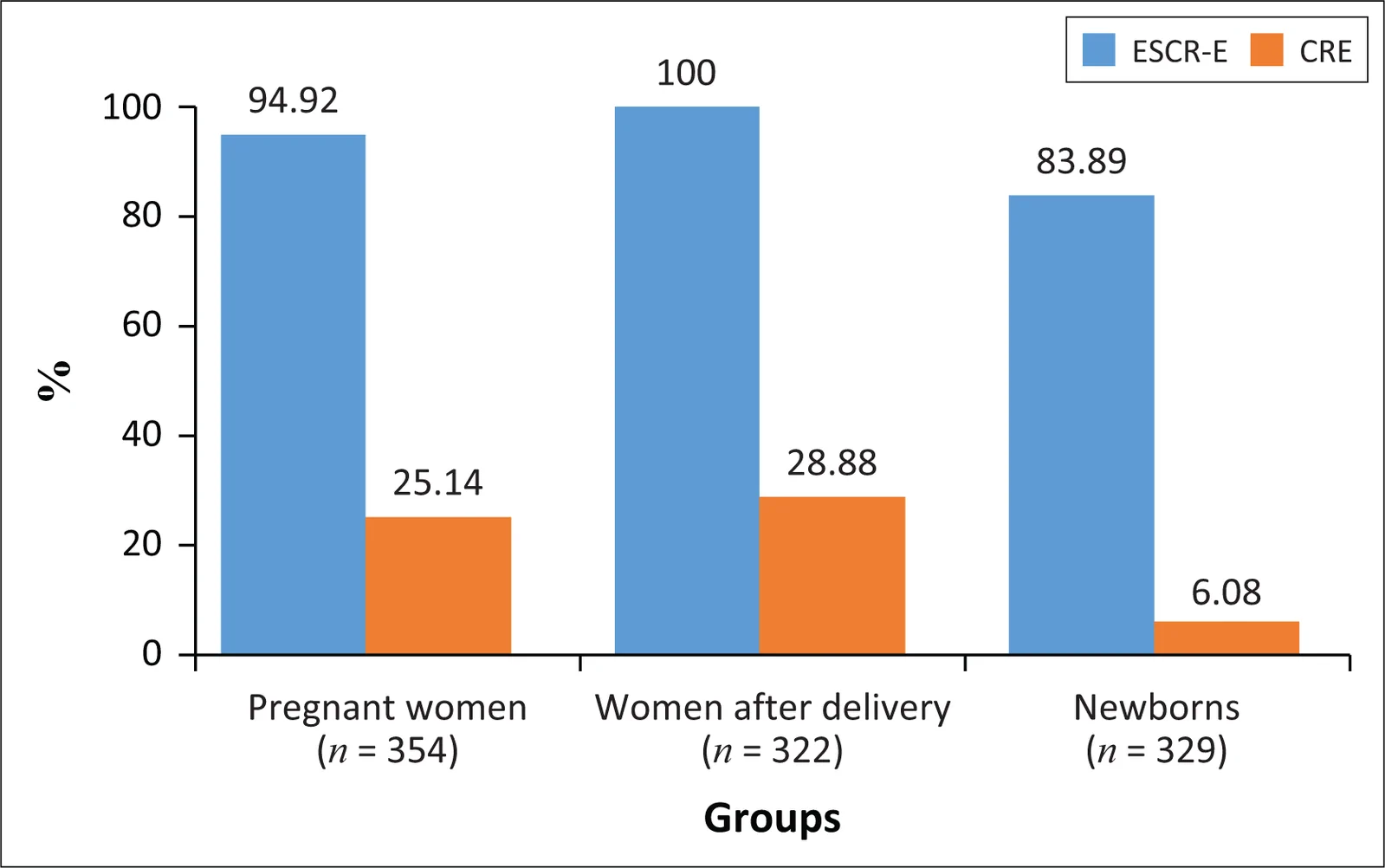
Faecal carriage rate of extended-spectrum cephalosporin-resistant *Enterobacterales* and carbapenem-resistant *Enterobacteriaceae* among pregnant women, women after delivery, and newborns, April 2023 to October 2024, South Kivu province, DR Congo.

### Resistance profiles of extended-spectrum cephalosporin-resistant *Enterobacterales* species and *Acinetobacter baumannii* isolated from pregnant women

In this study, antibiotic susceptibility testing was performed on isolates recovered from pregnant women. The *Enterobacterales* tested included *E. coli* (*n* = 96), *K. pneumoniae* (*n* = 88), *Klebsiella aerogenes* (*n* = 78), *Citrobacter freundii* (*n* = 18), *Proteus mirabilis* (*n* = 9), and *Salmonella enterica* (*n* = 22). In addition, *A. baumannii* (*n* = 25), a non-*Enterobacterales* Gram-negative species, was analysed separately (Table 1).

**TABLE 1:** Antibiotic resistance profiles of extended-spectrum cephalosporin-resistant *Enterobacterales* species in pregnant women (*N* = 336) April 2023 to October 2024, South Kivu province, DR Congo.

Antibiotics	*Escherichia coli* (*n* = 96)		*Klebsiella pneumoniae* (*n* = 88)		*Klebsiella aerogenes* (*n* = 78)		*Citrobacter freundii* (*n* = 18)		*Proteus mirabilis* (*n* = 9)		*Salmonella enterica* (*n* = 22)		*Acinetobacter baumannii* (*n* = 25)	
	*n*	%	*n*	%	*n*	%	*n*	%	*n*	%	*n*	%	*n*	%
Amoxicillin	95	98.9	88	100.0	78	100.0	18	100.0	9	100.0	22	100.0	25	100.0
Ticarcillin	91	94.8	87	98.9	76	97.4	18	100.0	9	100.0	21	95.5	25	100.0
Cefixime	86	89.6	83	94.3	73	93.6	17	94.4	9	100.0	20	90.9	25	100.0
Ciprofloxacin	82	85.4	81	92.0	69	88.5	15	83.3	7	77.8	19	86.4	25	100.0
Cefotaxime	89	92.7	84	95.5	74	94.9	17	94.4	9	100.0	21	95.5	25	100.0
Gentamicin	74	77.1	77	87.5	69	88.5	13	72.2	6	66.7	16	72.7	24	96.0
Amikacin	62	64.6	65	73.9	62	79.5	12	66.7	5	55.6	14	63.6	23	92.0
Chloramphenicol	78	81.3	73	83.0	66	84.6	13	72.2	6	66.7	18	81.8	25	100.0
Tétracycline	83	86.5	81	92.0	69	88.5	16	88.9	7	77.8	19	86.4	24	96.0
Ceftriaxone	88	91.7	85	96.6	72	92.3	17	94.4	9	100.0	21	95.5	25	100.0
Ceftazidime	91	94.8	84	95.5	73	93.6	17	94.4	9	100.0	21	95.5	25	100.0
Cefta–Tazobactam	0	0.0	0	0.0	0	0.0	0	0.0	0	0.0	0	0.0	0	0.0

The antibiogram results among extended-spectrum β-lactamase-producing *Enterobacterales* isolated from pregnant women reveal alarming resistance patterns. Nearly all isolates showed over 90% resistance to commonly used beta-lactams, including amoxicillin, ticarcillin, cefixime, cefotaxime, ceftriaxone, and ceftazidime. Ciprofloxacin, gentamicin, and tetracycline also showed high resistance rates, indicating limited therapeutic options. Notably, all strains were fully susceptible to the ceftazidime–tazobactam combination (0% resistance), suggesting potential presence of advanced β-lactamases beyond classic extended-spectrum β-lactamases. Amikacin retained relatively better activity but with declining efficacy.

High resistance rates were observed for several antibiotics commonly used in clinical practice. Among the *Enterobacterales*, resistance to amoxicillin was nearly universal, ranging from 98.9% in *E. coli* to 100% in the other species, while ticarcillin resistance ranged from 94.8% to 100%. Resistance to third-generation cephalosporins, including cefixime, cefotaxime, ceftriaxone, and ceftazidime, was consistently high, ranging from 89.6% to 100% among Enterobacterales. Ciprofloxacin resistance ranged from 77.8% to 100% among *Enterobacterales*. Aminoglycoside resistance was also substantial, with gentamicin resistance ranging from 66.7% (*P. mirabilis*) to 90.9% (*K. aerogenes*), and amikacin resistance between 55.6% and 88.6%. Resistance to chloramphenicol and tetracycline was generally above 70% (Table 1).

*Acinetobacter baumannii*, analysed separately, displayed similarly high resistance: 100% to amoxicillin and ticarcillin; 100% to cefotaxime, ceftriaxone, and cefixime; and high resistance to ciprofloxacin (100%) and aminoglycosides (gentamicin 96.0%, amikacin 92.0%) (Table 1).

Notably, none of the isolates, whether *Enterobacterales* or *A. baumannii*, showed resistance to the combination of ceftazidime and tazobactam (Table 1).

These findings reveal a multidrug-resistance pattern among *Enterobacterales* colonising pregnant women, with *A. baumannii* exhibiting a similar but separate MDR profile, which could complicate empirical treatment strategies (Table 1).

### Resistance profiles of extended-spectrum cephalosporin-resistant *Enterobacterales* species and *Acinetobacter baumannii* isolated among post-delivery women

The antibiotic susceptibility of isolates recovered from women at delivery was evaluated across nine bacterial species. The *Enterobacterales* included *C. freundii* (*n* = 18), *E. coli* (*n* = 87), *K. aerogenes* (*n* = 67), *K. pneumoniae* (*n* = 71), *Morganella morganii* (*n* = 9), *P. mirabilis* (*n* = 14), *S. enterica* (*n* = 22), and *Serratia marcescens* (*n* = 1). In addition, *A. baumannii* (*n* = 34) was analysed separately (Table 2).

**TABLE 2:** Resistance rates of extended-spectrum cephalosporin-resistant *Enterobacterales* species isolated in delivery women (*N* = 323) April 2023 to October 2024, South Kivu province, DR Congo.

Antibiotics	*Acinetobacter baumannii* (*n* = 34)		*Citrobacter freundii* (*n* = 18)		*Escherichia coli* (*n* = 87)		*Klebsiella aerogenes* (*n* = 67)		*Klebsiella pneumoniae* (*n* = 71)		*Morganella morgani* (*n* = 9)		*Proteus mirabilis* (*n* = 14)		*Salmonella enterica* (*n* = 22)		*Serratia marcescens* (*n* = 1)	
	*n*	%	*n*	%	*n*	%	*n*	%	*n*	%	*n*	%	*n*	%	*n*	%	*n*	%
Amoxicilline	34	100.0	17	94.0	83	95.0	65	97.0	69	97.0	9	100.0	13	93.0	21	95.0	1	100.0
Ticarcilline	34	100.0	16	89.0	84	97.0	66	99.0	70	99.0	9	100.0	14	100.0	21	95.0	1	100.0
Cefixime	33	97.0	16	89.0	82	94.0	65	97.0	70	99.0	9	100.0	14	100.0	21	95.0	1	100.0
Ciprofloxacin	29	85.0	12	67.0	61	70.0	43	64.0	41	58.0	8	89.0	12	86.0	14	64.0	1	100.0
Cefotaxime	33	97.0	16	89.0	79	91.0	61	91.0	65	92.0	9	100.0	14	100.0	20	91.0	1	100.0
Gentamicine	31	91.0	13	72.0	65	75.0	50	75.0	52	73.0	8	89.0	11	79.0	15	68.0	1	100.0
Amikacine	30	88.0	12	67.0	57	66.0	47	70.0	43	61.0	8	89.0	10	71.0	14	64.0	1	100.0
Chloramphenicol	26	76.0	11	61.0	46	53.0	42	63.0	44	62.0	6	67.0	9	64.0	12	55.0	1	100.0
Tétracycline	29	85.0	12	67.0	62	71.0	46	69.0	46	65.0	7	78.0	11	79.0	14	64.0	1	100.0
Ceftriaxone	33	97.0	16	89.0	79	91.0	61	91.0	65	92.0	9	100.0	14	100.0	20	91.0	1	100.0
Ceftazidime	33	97.0	16	89.0	79	91.0	61	91.0	65	92.0	9	100.0	14	100.0	20	91.0	1	100.0
Ceftazidime–Tazobactam	0	0.0	0	0.0	0	0.0	0	0.0	0	0.0	0	0.0	0	0.0	0	0.0	0	0.0

The resistance profiles of extended-spectrum cephalosporin-resistant *Enterobacterales* isolated from delivery women reveal widespread and concerning multidrug resistance. Almost all isolates, including *E. coli, K. pneumoniae, A. baumannii*, and *P. mirabilis*, exhibited over 90% resistance to first-line beta-lactams and third-generation cephalosporins. Ciprofloxacin, gentamicin, and amikacin showed slightly better efficacy but still with high resistance rates (often > 60%). Notably, all isolates were susceptible to ceftazidime–tazobactam (0% resistance), indicating the possible circulation of advanced resistance mechanisms.

Among *Enterobacterales*, resistance to amoxicillin and ticarcillin was nearly universal, ranging from 93% to 100%. Third-generation cephalosporins – including cefixime, cefotaxime, ceftriaxone, and ceftazidime – showed high resistance rates, generally exceeding 89%. Ciprofloxacin resistance varied from 58% in *K. pneumoniae* to 95% in *C. freundii*, with intermediate values observed in other species. Aminoglycoside resistance was substantial, with gentamicin resistance ranging from 68% to 88%, and amikacin resistance from 61% to 86%. Resistance to chloramphenicol and tetracycline was also high, generally between 53% and 79% (Table 2).

*Acinetobacter baumannii*, analysed separately from *Enterobacterales*, displayed similarly high resistance: 100% to amoxicillin and ticarcillin, 97% to cefixime, cefotaxime, ceftriaxone, and ceftazidime, and high resistance to ciprofloxacin (95%) and aminoglycosides (gentamicin 91%, amikacin 89%) (Table 2).

Notably, none of the isolates – whether *Enterobacterales* or *A. baumannii* – demonstrated resistance to the combination of ceftazidime and tazobactam (Table 2).

Overall, these results reveal a high prevalence of multidrug resistance among *Enterobacterales* colonising women at delivery, with *A. baumannii* exhibiting a separate but comparable MDR profile, representing a significant challenge for empirical antimicrobial therapy in this population (Table 2).

### Resistance profiles of extended-spectrum cephalosporin-resistant *Enterobacterales* species isolated among newborns

Among the 276 ESCR-E isolates recovered from newborns, three species predominated: *E. coli* (*n* = 120), *K. aerogenes* (*n* = 82), and *K. pneumoniae* (*n* = 74). Resistance to first-line antibiotics, including amoxicillin and ticarcillin, was universal across all isolates (100%), and all strains also showed complete resistance to cefixime (Table 3).

**TABLE 3:** Antibiotic resistance profiles of extended-spectrum cephalosporin-resistant Enterobacterales species isolated among newborns (*N* = 276) April 2023 to October 2024, South Kivu province, DR Congo.

Antibiotics	*Escherichia coli* (*n* = 120)		*Klebsiella aerogenes* (*n* = 82)		*Klebsiella pneumoniae* (*n* = 74)	
	*n*	%	*n*	%	*n*	%
Amoxicillin	120	100.0	82	100.0	74	100.0
Ticarcillin	120	100.0	82	100.0	74	100.0
Cefixime	120	100.0	82	100.0	74	100.0
Ciprofloxacin	76	63.3	48	58.5	49	66.2
Cefotaxime	107	89.2	72	87.8	66	89.2
Gentamicin	113	94.2	78	95.1	70	94.6
Amikacin	34	28.3	18	22.0	20	27.0
Chloramphenicol	100	83.3	74	90.2	65	87.8
Tetracycline	102	85.0	71	86.6	67	90.5
Ceftriaxone	112	93.3	76	92.7	70	94.6
Ceftazidime	112	93.3	76	92.7	69	93.2
Ceftazidime–Tazobactam	0	0.0	0	0.0	0	0.0

The antibiotic resistance data among newborns reveal an alarming prevalence of multidrug-resistant extended-spectrum cephalosporin-resistant *Enterobacterales* strains. All isolates of *E. coli, K. aerogenes*, and *K. pneumoniae* showed 100% resistance to amoxicillin, ticarcillin, and cefixime. High resistance was also seen for cefotaxime (88.8%), ceftriaxone (93.5%), and ceftazidime (93.1%). Gentamicin showed limited effectiveness, with resistance over 94%, while amikacin retained some activity (only 26.1% resistance). all isolates were susceptible to ceftazidime–tazobactam (0% resistance).

High levels of resistance were also observed to third-generation cephalosporins: 93.5% for ceftriaxone, 93.1% for ceftazidime, and 88.8% for cefotaxime. Ciprofloxacin resistance was moderate, affecting 62.7% of isolates overall, with slightly higher rates among *K. pneumoniae* (66.2%) and *E. coli* (63.3%) (Table 3).

Resistance to aminoglycosides was variable. While gentamicin resistance was alarmingly high at 94.6%, amikacin remained relatively effective, with only 26.1% of isolates resistant. Resistance to chloramphenicol (86.6%) and tetracycline (87.0%) was also consistently elevated across species (Table 3).

Notably, none of the ESCR-E isolates was resistant to the combination of ceftazidime–tazobactam (0%), indicating its potential retained efficacy (Table 3).

These findings highlight a worrying burden of MDR *Enterobacterales* among neonatal colonisers, with limited therapeutic options remaining, especially for aminoglycosides and third-generation cephalosporins (Table 3).

### Resistance profiles of carbapenem-resistant *Enterobacteriaceae* species and *Acinetobacter baumannii* isolated among pregnant women

A total of 89 carbapenem-resistant isolates were obtained from pregnant women. Among them, the *Enterobacterales* included *E. coli* (*n* = 42), *K. pneumoniae* (*n* = 14), and *K. aerogenes* (*n* = 8). In addition, *A. baumannii* (*n* = 25) was also analysed (Table 4).

**TABLE 4:** Resistance associated by carbapenem-resistant *Enterobacteriaceae* species isolated among pregnant women April 2023 to October 2024, South Kivu province, DR Congo.

Antibiotics	*Escherichia coli* (*n* = 42)		*Acinetobacter baumannii* (*n* = 25)		*Klebsiella pneumoniae* (*n* = 14)		*Klebsiella aerogenes* (*n* = 8)	
	*n*	%	*n*	%	*n*	%	*n*	%
Amoxicillin	42	100.0	25	100.0	14	100.0	8	100.0
Ticarcillin	42	100.0	25	100.0	14	100.0	8	100.0
Cefixime	42	100.0	25	100.0	14	100.0	8	100.0
Ciprofloxacin	34	81.0	25	100.0	7	50.0	8	100.0
Cefotaxime	42	100.0	25	100.0	14	100.0	8	100.0
Gentamicin	42	100.0	25	100.0	14	100.0	8	100.0
Amikacin	26	62.0	25	100.0	14	100.0	8	100.0
Chloramphenicol	29	69.0	25	100.0	14	100.0	8	100.0
Tetracycline	28	67.0	25	100.0	14	100.0	8	100.0
Ceftriaxone	42	100.0	25	100.0	14	100.0	8	100.0
Ceftazidime	42	100.0	25	100.0	14	100.0	8	100.0
Ceftazidime–Tazobactam	0	0.0	0	0.0	0	0.0	0	0.0
Ertapenem	42	100.0	25	100.0	14	100.0	8	100.0
Imipenem	39	93.0	21	84.0	14	100.0	8	100.0
Meropenem	40	95.0	25	100.0	6	43.0	7	88.0

Carbapenem-resistant *Enterobacteriaceae* strains isolated from pregnant women showed 100% resistance to most β-lactams, including amoxicillin, cefotaxime, and ceftriaxone. However, all isolates were 100% **susceptible** to ceftazidime–tazobactam, suggesting these may still be effective options. *Klebsiella pneumoniae* showed notably lower resistance to meropenem (43%) compared to other species. Ciprofloxacin resistance varied, with only 50% of *K. pneumoniae* isolates resistant.

Across all *Enterobacterales* isolates, universal resistance (100%) was observed to β-lactam antibiotics, including amoxicillin, ticarcillin, cefixime, cefotaxime, ceftriaxone, and ceftazidime. Full resistance to ertapenem was also observed in *Enterobacterales* isolates, confirming their CRE phenotype. Ciprofloxacin resistance among *Enterobacterales* varied: 50% in *K. pneumoniae*, 81% in *E. coli*, and 100% in *K. aerogenes*. Gentamicin resistance reached 100% in all *Enterobacterales*, whereas amikacin resistance was lower in *E. coli* (62%) but 100% in *K. pneumoniae* and *Klebsiella aerogenes*. Resistance to chloramphenicol and tetracycline among *Enterobacterales* was 69% and 67% in *E. coli*, and 100% in *K. pneumoniae* and *K. aerogenes*. Resistance to imipenem and meropenem among *Enterobacterales* ranged from 84% to 100% and 43% to 100%, with the lowest meropenem resistance observed in *K. pneumoniae* (43%) (Table 4).

*Acinetobacter baumannii*, analysed separately, exhibited universal resistance to β-lactams (amoxicillin, ticarcillin, cefixime, cefotaxime, ceftriaxone, ceftazidime), ertapenem, gentamicin, amikacin, chloramphenicol, and tetracycline. Ciprofloxacin and carbapenem resistance were also high, with 100% of isolates resistant to ciprofloxacin, imipenem, and meropenem (Table 4).

### Resistance profiles of carbapenem-resistant *Enterobacteriaceae* species isolated among post-delivery women

Among the 93 carbapenem-resistant isolates collected from women at delivery, the *Enterobacterales* included *E. coli* (*n* = 43), *K. pneumoniae* (*n* = 19), and *Klebsiella aerogenes* (*n* = 8). *Acinetobacter baumannii* (*n* = 23), a non-*Enterobacterales* Gram-negative species, was analysed separately (Table 5).

**TABLE 5:** Resistance associated by carbapenem-resistant *Enterobacteriaceae* species isolated among delivery women April 2023 to October 2024, South Kivu province, DR Congo.

Antibiotics	*Escherichia coli* (*n* = 43)		*Acinetobacter baumannii* (*n* = 23)		*Klebsiella pneumoniae* (*n* = 19)		*Klebsiella aerogenes* (*n* = 8)	
	*n*	%	*n*	%	*n*	%	*n*	%
Amoxicillin	43	100.0	23	100.0	19	100.0	8	100.0
Ticarcillin	43	100.0	23	100.0	19	100.0	8	100.0
Cefixime	43	100.0	23	100.0	19	100.0	8	100.0
Ciprofloxacin	35	81.4	23	100.0	19	100.0	7	87.5
Cefotaxime	43	100.0	23	100.0	19	100.0	8	100.0
Gentamicin	41	95.3	23	100.0	18	94.7	8	100.0
Amikacin	35	81.4	23	100.0	17	89.5	8	100.0
Chloramphenicol	34	79.1	23	100.0	17	89.5	8	100.0
Tetracycline	36	83.7	23	100.0	18	94.7	8	100.0
Ceftriaxone	43	100.0	23	100.0	19	100.0	8	100.0
Ceftazidime	43	100.0	23	100.0	19	100.0	8	100.0
Ceftazidime–Tazobactam	0	0.0	0	0.0	0	0.0	0	0.0
Ertapenem	43	100.0	23	100.0	19	100.0	8	100.0
Imipenem	39	90.7	23	100.0	17	89.5	6	75.0
Meropenem	43	100.0	23	100.0	19	100.0	8	100.0

Carbapenem-resistant *Enterobacteriaceae* isolates from delivery women showed 100% resistance to most beta-lactams, including amoxicillin, ticarcillin, and cefotaxime. However, **all strains were fully susceptible (0% resistance)** to ceftazidime–tazobactam, offering possible treatment alternatives. Ciprofloxacin and aminoglycosides (gentamicin and amikacin) showed variable resistance, especially in *E. coli*. Imipenem resistance was variable, with 75.0% resistance among *K. aerogenes*.

Across all *Enterobacterales* isolates, resistance was universal (100%) to key β-lactam antibiotics, including amoxicillin, ticarcillin, cefixime, cefotaxime, ceftriaxone, and ceftazidime. Complete resistance to the carbapenems ertapenem and meropenem was also observed. Ciprofloxacin resistance among *Enterobacterales* ranged from 81.4% in *E. coli* to 87.5% in *Klebsiella aerogenes*, and gentamicin resistance ranged from 94.7% to 100%. Amikacin resistance was slightly lower in *E. coli* (81.4%) and *K. pneumoniae* (89.5%). Resistance to chloramphenicol and tetracycline ranged from 79.1% to 100%, with *E. coli* exhibiting the lowest rates. Resistance to imipenem varied among *Enterobacterales*, with 90.7% in *E. coli* and 75% in *K. aerogenes*, whereas *K. pneumoniae* showed complete resistance (100%) (Table 5).

*Acinetobacter baumannii* exhibited universal resistance to all β-lactams (amoxicillin, ticarcillin, cefixime, cefotaxime, ceftriaxone, ceftazidime), ertapenem, meropenem, gentamicin, amikacin, chloramphenicol, tetracycline, and ciprofloxacin. Resistance to imipenem and meropenem was complete (100%) in all *A. baumannii* isolates (Table 5).

Notably, all isolates – whether *Enterobacterales* or *A. baumannii* – remained fully susceptible to ceftazidime–tazobactam (0% resistance), indicating retained activity of this combination against CRE isolates from women at delivery (Table 5).

### Resistance profiles of carbapenem-resistant *Enterobacteriaceae* species isolated among newborns

Among the 20 CRE isolates obtained from neonates, *E. coli* (*n* = 11), *Klebsiella aerogenes* (*n* = 5), and *K. pneumoniae* (*n* = 4) were identified. All isolates from the three species showed 100% resistance to first-line β-lactam antibiotics, including amoxicillin, ticarcillin, cefixime, cefotaxime, ceftriaxone, and ceftazidime (Table 6).

**TABLE 6:** Antibiotic resistance profile of carbapenem-resistant *Enterobacteriaceae* isolates from neonates (*N* = 20) April 2023 to October 2024, South Kivu province, DR Congo.

Antibiotics	*Escherichia coli* (*n* = 11)		*Klebsiella aerogenes* (*n* = 5)		*Klebsiella pneumoniae* (*n* = 4)	
	*n*	%	*n*	%	*n*	%
Amoxicillin	11	100.0	5	100.0	4	100.0
Ticarcillin	11	100.0	5	100.0	4	100.0
Cefixime	11	100.0	5	100.0	4	100.0
Ciprofloxacin	9	81.8	4	80.0	4	100.0
Cefotaxime	11	100.0	5	100.0	4	100.0
Gentamicin	11	100.0	5	100.0	4	100.0
Amikacin	11	100.0	4	80.0	3	75.0
Chloramphenicol	10	90.9	5	100.0	3	75.0
Tetracycline	10	90.9	5	100.0	3	75.0
Ceftriaxone	11	100.0	5	100.0	4	100.0
Ceftazidime	11	100.0	5	100.0	4	100.0
Ceftazidime–Tazobactam	0	0.0	0	0.0	0	0.0
Ertapenem	10	90.9	5	100.0	4	100.0
Imipenem	10	90.9	4	80.0	4	100.0
Meropenem	11	100.0	4	80.0	4	100.0

Carbapenem-resistant *Enterobacteriaceae* isolates from newborns exhibited complete resistance (100%) to first-line antibiotics such as amoxicillin, ticarcillin, and cefotaxime. All isolates remained fully susceptible (0% resistance) to ceftazidime–tazobactam, suggesting retained efficacy. Carbapenem resistance remained high, ranging from 80.0% to 100% across the three species. Ciprofloxacin and chloramphenicol showed moderate resistance, particularly in *K. pneumoniae*. These results underscore the therapeutic challenge posed by carbapenem-resistant *Enterobacteriaceae* in neonates and the need for careful antibiotic selection.

Resistance to ciprofloxacin was complete in *K. pneumoniae*, and high in *E. coli* (81.8%) and *Klebsiella aerogenes* (80.0%). Likewise, all isolates were resistant to gentamicin. Resistance to amikacin was slightly lower, with *K. pneumoniae* (75.0%) and *K. aerogenes* (80.0%) showing more susceptibility than *E. coli* (100%) (Table 6).

Regarding other antibiotic classes, resistance to chloramphenicol and tetracycline ranged from 75.0% to 100%, with *K. pneumoniae* isolates again showing relatively lower resistance. Notably, sensitivity to the combination ceftazidime–tazobactam was universal across all isolates (100%) (Table 6).

Resistance to carbapenems was also very high. All *K. pneumoniae* and *K. aerogenes* isolates were resistant to ertapenem, imipenem, and meropenem. Among *E. coli* isolates, resistance to ertapenem was 90.9%, while both imipenem and meropenem resistance were also 90.9% (Table 6).

## Discussion

### High rate of extended-spectrum cephalosporin-resistant *Enterobacterales* and carbapenem-resistant *Enterobacteriaceae* faecal carriage among pregnant women, women after delivery, and newborns

In our study, we observed an alarmingly high faecal carriage of ESCR-E among pregnant women 94.9% during pregnancy and 100% at delivery, as well as a high rate (83.9%) among their newborns. The carriage of CRE was also substantial in mothers (25.1% in pregnant women; 28.9% at delivery) and notable in newborns (6.1%). These prevalences markedly exceed the rates generally reported in the literature, highlighting a potentially severe public health issue in our setting. The findings have been reported in a separate publication

### Resistance profile of extended-spectrum cephalosporin-resistant *Enterobacterales*

This study investigated the AMR patterns of ESCR-E isolated from pregnant women, postpartum mothers, and their newborns in South Kivu, eastern DRC. Our findings highlight a critical public health threat, with widespread multidrug resistance compromising treatment options in maternal and neonatal care.

Resistance to third-generation cephalosporins – cefotaxime, ceftriaxone, ceftazidime, and cefixime – was uniformly high, exceeding 90% in all major species including *E. coli, K. pneumoniae, A. baumannii*, and *K. aerogenes*. This resistance profile is consistent with an ESBL phenotype, likely dominated by CTX-M-type enzymes, which are well documented in both hospital and community settings across sub-Saharan Africa.^23^

Beyond β-lactams, resistance to fluoroquinolones was also alarmingly high. Over 85% of *K. pneumoniae, A. baumannii*, and *E. coli* isolates were resistant to ciprofloxacin, suggesting co-selection by means of plasmids carrying multiple resistance determinants. Such plasmids often harbor genes such as *qnr, aac(6’)-Ib-cr*, and *qepA*, which confer fluoroquinolone resistance and are frequently co-located with ESBL genes.^24,25^ This co-resistance pattern reflects both poor antibiotic stewardship and widespread empirical use of broad-spectrum agents without microbiological confirmation, a scenario common in resource-limited settings.

Similarly, high resistance levels were observed for aminoglycosides such as gentamicin, as well as older agents such as tetracycline and chloramphenicol. The diminished efficacy of these traditionally first-line antibiotics severely restricts the therapeutic options available for treating neonatal and postpartum infections, potentially increasing morbidity and mortality in these vulnerable populations.

Interestingly, ceftazidime–tazobactam remained fully active against all ESCR-E isolates in this study. This suggests either limited prior use or absence of resistance mechanisms targeting β-lactam/β-lactamase inhibitor combinations in the region. As such, this drug may serve as a critical therapeutic option in severe infections, particularly where carbapenems are inaccessible. However, caution is warranted: resistance to this combination has emerged rapidly elsewhere because of misuse, and over-reliance could threaten its effectiveness.^26,27^

Our findings are consistent with other studies from sub-Saharan Africa. In Tanzania, over 95% of neonatal *E. coli* isolates were resistant to third-generation cephalosporins, and over 80% to ciprofloxacin.^28,29^ In Nigeria, maternal ESBL-E colonisation was strongly associated with neonatal ESBL-E colonisation,^30^ and in Ethiopia, high ESBL production rates with fluoroquinolone co-resistance were also reported.^31^ This convergence in resistance profiles across East and West Africa underscores shared systemic drivers – poor antimicrobial regulation, limited diagnostics, and inadequate infection prevention in maternity wards.

Such conditions foster the propagation of ESCR-E and other MDR organisms, exacerbating already fragile healthcare systems. The presence of these resistant pathogens not only limits treatment efficacy but also heightens the risk of nosocomial transmission and therapeutic failure. This emphasises the need for coordinated interventions, including improved hygiene protocols, enhanced antimicrobial stewardship, and community-level awareness campaigns. These efforts must be reinforced by robust laboratory-based surveillance systems capable of tracking resistance trends and guiding empirical therapy in real time.

### Resistance patterns in carbapenem-resistant *Enterobacteriaceae* isolates

In addition to ESCR-E, this study identified an equally concerning resistance profile among CRE isolates. All CRE isolates exhibited universal resistance to penicillins and third-generation cephalosporins, strongly suggesting the presence of multiple β-lactamases, including both ESBLs and carbapenemases.^32^

The most likely mechanisms include carbapenemase genes such as *bla_KPC, bla_NDM, bla_VIM, bla_IMP*, and *bla_OXA-48-like*, which encode *KPC, NDM, VIM, IMP* and *OXA-48* carbapenemase enzymes. In Africa, *NDM* and *OXA-48*-like carbapenemases are particularly prevalent and have been reported in neonatal outbreaks.^33,34^

These enzymes confer broad-spectrum resistance and are typically plasmid-borne, facilitating horizontal gene transfer within healthcare environments.

All CRE isolates in this study also showed high resistance to ciprofloxacin and gentamicin, particularly among *A. baumannii, K. aerogenes*, and *K. pneumoniae*. This co-resistance likely reflects plasmid-mediated gene clusters, which include fluoroquinolone resistance determinants such as *qnr* and aminoglycoside-modifying enzymes such as *aac(6’)-Ib*, often accompanied by efflux pump overexpression.^35,36^ Resistance to additional agents – tetracycline, amikacin, and chloramphenicol – further classifies these strains as MDR and highlights the limited therapeutic landscape in South Kivu.

Remarkably, no phenotypic resistance to ceftazidime–tazobactam was detected among the CRE isolates under the testing conditions used in this study. However, previous in vitro studies have reported variable activity of this combination against Gram-negative bacteria, depending on the bacterial species and underlying β-lactamase mechanisms.^37,38^ Because molecular characterisation of the isolates was not performed, the mechanisms underlying the observed activity could not be determined. Therefore, these findings should be interpreted cautiously, and phenotypic confirmation remains essential before empirical use.

Colistin, often considered the last resort for CRE infections, was not tested in this study. However, its use remains extremely rare in the region, and the absence of routine testing for plasmid-mediated resistance (e.g. *mcr* genes) represents a critical gap. The global spread of *mcr*-positive strains is deeply concerning because of the risk of horizontal gene transfer and community-level dissemination.^39,40^

The emergence of CRE in high-risk populations such as mothers and neonates demands urgent attention. These infections are associated with high mortality, prolonged hospital stays, and significant economic burden.^32,41^ Yet in much of sub-Saharan Africa, surveillance of carbapenem resistance is either weak or nonexistent. Laboratory testing for carbapenemases is not routine, leading to underestimation of prevalence and missed opportunities for containment.^42^

To address this challenge, investments in diagnostic infrastructure are essential, alongside Infection Prevention and Control training and strict regulation of antibiotic use. The limited but preserved susceptibility to ceftazidime–tazobactam must be safeguarded through targeted, rational use supported by local susceptibility data. The implementation of national AMR control programmes, coupled with international collaboration, is crucial for early detection and containment of CRE and other high-risk pathogens.

### Study limitations

This study has several important limitations. Firstly, its cross-sectional design limits the ability to draw causal inferences or assess temporal resistance trends. Secondly, the absence of molecular analysis precluded confirmation of the genetic basis of resistance, such as the presence of *bla_CTX-M, bla_NDM*, or *mcr* genes. Thirdly, susceptibility testing did not include colistin, which is a key last-resort antibiotic in the context of carbapenem resistance. Fourth, the study was limited to selected healthcare facilities in South Kivu and may not reflect resistance patterns across other regions of the DRC. Fifth, because of resource constraints, not all antibiotics could be tested, and only phenotypic methods were employed, which may fail to detect certain resistance mechanisms. Finally, minimum inhibitory concentrations were not determined, limiting the precision in assessing the degree of bacterial susceptibility or resistance.

### Conclusion

Our findings reveal a high prevalence of ESCR-E and CRE carriage among pregnant women and neonates in South Kivu, indicating a concerning burden of MDR *Enterobacterales* in the maternal–neonatal ecosystem. The universal resistance to third-generation cephalosporins, alongside elevated resistance to aminoglycosides, fluoroquinolones, and other agents, severely restricts therapeutic options in this resource-limited context. The preserved susceptibility to ceftazidime–tazobactam offers a narrow but critical treatment opportunity that must be safeguarded.

Strengthening antimicrobial stewardship, improving access to microbiology diagnostics, and reinforcing infection prevention and control are urgently required to mitigate potential mother-to-child transmission and broader community dissemination. These interventions should be guided by local surveillance data and adapted to the operational realities of low-resource healthcare systems.

Given the impact of antimicrobial exposure on intestinal microbiota, complementary strategies such as targeted probiotics may support decolonisation and bolster current infection prevention and control efforts. Future studies assessing the dynamics of acquisition and transmission within mother–newborn pairs are warranted.

Sustained integration of AMR surveillance into maternal and neonatal care, aligned with national and global action plans, is essential to protect vulnerable populations and preserve the effectiveness of life-saving antibiotics.
